# Graphene Oxide Functional Nanohybrids with Magnetic Nanoparticles for Improved Vectorization of Doxorubicin to Neuroblastoma Cells

**DOI:** 10.3390/pharmaceutics11010003

**Published:** 2018-12-22

**Authors:** Luigi Lerra, Annafranca Farfalla, Beatriz Sanz, Giuseppe Cirillo, Orazio Vittorio, Florida Voli, Marion Le Grand, Manuela Curcio, Fiore Pasquale Nicoletta, Anna Dubrovska, Silke Hampel, Francesca Iemma, Gerardo F. Goya

**Affiliations:** 1 Children’s Cancer Institute, Lowy Cancer Research Centre, UNSW Sydney, NSW 2031, Australia; LLerra@ccia.org.au (L.L.); OVittorio@ccia.org.au (O.V.); FVoli@ccia.org.au (F.V.); MLeGrand@ccia.org.au (M.L.G.); 2 Department of Pharmacy Health and Nutritional Science, University of Calabria, 87036 Rende (CS), Italy; annafranca.farfalla@gmail.com (A.F.); manuela.curcio@unical.it (M.C.); fiore.nicoletta@unical.it (F.P.N.); francesca.iemma@unical.it (F.I.); 3 nB nanoSacale Biomagnetics SL, 50012 Zaragoza, Spain; bsanzsague@gmail.com; 4 ARC Centre of Excellence for Convergent BioNano Science and Technology, Australian Centre for NanoMedicine, UNSW Sydney, NSW 2052, Australia; 5 School of Women’s and Children’s Health, Faculty of Medicine, UNSW Sydney, NSW 2052, Australia; 6 OncoRay—National Center for Radiation Research in Oncology, Faculty of Medicine and University Hospital Carl Gustav Carus, Technische Universität Dresden, 01307 Dresden, Germany; Anna.Dubrovska@OncoRay.de; 7 German Cancer Research Center (DKFZ), 69120 Heidelberg, Germany; 8 German Cancer Consortium (DKTK), partner site Dresden, 01307 Dresden, Germany; 9 Helmholtz-Zentrum Dresden-Rossendorf, Institute of Radiooncology-Oncoray, 01307 Dresden, Germany; 10 Leibniz Institute of Solid State and Material Research Dresden, 01069 Dresden, Germany; s.hampel@ifw-dresden.de; 11 Institute of Nanoscience of Aragon (INA), Department of Condensed Matter Physics, University of Zaragoza, 50018 Zaragoza, Spain; goya@unizar.es

**Keywords:** graphene oxide, iron oxide nanoparticles, magnetic targeting, nanohybrids, synergism

## Abstract

With the aim to obtain a site-specific doxorubicin (DOX) delivery in neuroblastoma SH-SY5Y cells, we designed an hybrid nanocarrier combining graphene oxide (GO) and magnetic iron oxide nanoparticles (MNPs), acting as core elements, and a curcumin–human serum albumin conjugate as functional coating. The nanohybrid, synthesized by redox reaction between the MNPs@GO system and albumin bioconjugate, consisted of MNPs@GO nanosheets homogeneously coated by the bioconjugate as verified by SEM investigations. Drug release experiments showed a pH-responsive behavior with higher release amounts in acidic (45% at pH 5.0) vs. neutral (28% at pH 7.4) environments. Cell internalization studies proved the presence of nanohybrid inside SH-SY5Y cytoplasm. The improved efficacy obtained in viability assays is given by the synergy of functional coating and MNPs constituting the nanohybrids: while curcumin moieties were able to keep low DOX cytotoxicity levels (at concentrations of 0.44–0.88 µM), the presence of MNPs allowed remote actuation on the nanohybrid by a magnetic field, increasing the dose delivered at the target site.

## 1. Introduction

Patients with neuroblastoma, the third most common type of extracranial solid cancer in the pediatric population [1], suffer for the severe acute and chronic toxicity of commonly employed therapeutics (e.g., vinca alkaloids, platinum drugs, and anthracyclines) due to the nonspecific in vivo distribution and their serious side effects [2,3]. The intravenous administration of conventional chemotherapy does not distinguish between cancerous and healthy cells and, especially in the case of childhood tumors, targeted and less toxic therapies are urgently required in order to improve the quality of life and reduce the lifelong health issues for the survivors [4,5]. To this regard, a great hope lies in the rising synergy between biomedicine and nanotechnology [6] with the possibility to develop delivery vehicles able to localize the delivery of payload in the tumor site [7,8].

In this work, we combined graphene oxide (GO), iron oxide nanoparticles (MNPs), and a new human serum albumin–curcumin conjugate (C@HSA) for the fabrication of a novel multifunctional nanohybrid (C@HSA-MNPs@rGO) to spatially control the vectorization of doxorubicin (DOX) to neuroblastoma SH-SY5Y cells. Due to its specific features, each component was observed to contribute to the performance of the final nanohybrid: (1) MNPs act as a targeting element [9,10,11]; (2) GO enhances the drug loading capability and makes the release profile prolonged over time [12,13,14,15]; and (3) immobilized curcumin (CUR) in the functional coating synergizes the drug cytotoxicity [16,17,18].

Due to their capability to interact with remote magnetic fields, magnetic nanoparticles are used in a growing number of strategies to actuate on biological systems for both diagnostic and therapeutic purposes [19,20,21,22]. Similarly, the use of GO-based nanomaterials as cargo bays for drug vectorization has grown rapidly in the past several years, by virtue of its unique structural and physic-chemical properties, excellent biocompatibility, large surface area, and low cost [23,24,25,26,27]. We previously proved that the coating of carbon nanostructures with polyphenol–polymer conjugates constituted an efficient drug delivery system whose anticancer activity is related to either the loaded drug or the carrier itself [28,29]. The resulting nanohybrid described here profits from two main concurrent effects: (1) the synergistic mechanism of polyphenols associated with conventional anticancer drugs [30,31], and (2) the possibility to finely tune the drug release according to the carrier properties [32,33].

The experimental protocol involved the synthesis of MNPs stabilized with polyethyleneimine, and their subsequent mounting on GO sheets. Thus, MNPs@GO was loaded with doxorubicin and finally coated with a C@HSA to obtain the final nanohybrid, characterized in terms of physico chemical and magnetic properties and in vitro anticancer performance, providing new effective opportunities for targeted drug delivery.

## 2. Materials and Methods

### 2.1. Preparation of Graphene Oxide

A modified Hummers method was employed for the preparation of GO [34]. One gram of graphite (99.99%, 200 mesh), exfoliated by grinding with 50.0 g NaCl for 10 min, freed from NaCl by dissolution in distilled water (40 °C) and recovered by filtration using a 450 nm porous TEFLON filter paper, was mixed with 23 mL H_2_SO_4_ (96% *w*/*w*) overnight to allow graphite layers being intercalated by H_2_SO_4_.

The oxidization process was performed by adding 3.0 g KMnO_4_ over 3 h under constant stirring, and then the suspension was sonicated for 3 h and continuously stirred for 30 min at 35 °C and 45 min at 50 °C, respectively. Then, the suspension was added with distilled water (46 mL) and stirred for 45 min under reflux. After cooling to room temperature, distilled water (140 mL) and H_2_O_2_ (10 mL, 30%) were added to reduce the residual permanganate and manganese dioxide to manganese sulfate. The resulting material, filtered and washed 5 times with 5% HCl and distilled water to remove any reaction by-products, was cracked in water using a horn-tipped ultrasonic probe at 28 W for 2 h, in order to reduce the dimensions of the exfoliated GO sheets in terms of both lateral width and height. Uniformly sized GO particles (100 nm) were obtained via sucrose density gradient centrifugation. In a standard procedure, GO particles (335 mL) were added on the top of a gradient sucrose solutions (20–60% *w*/*v*), gently dropped into the bottom of centrifuged tube. The tube was directly centrifuged under controlled conditions (6000 g for 5 min) using Beckman Coulter, Allegra 64 R centrifuge, and the GO recovered by rinsing thoroughly with distilled water to remove sucrose.

All chemicals were from Merck KGaA (Darmstadt, Germany) for synthesis.

### 2.2. Synthesis of Magnetic Nanoparticles

The poly(ethyleneimine) (PEI)-coated magnetic nanoparticles (MNPs) were synthesized following the modified oxidative hydrolysis method [35]. The main steps of this protocol are as follows: a solution containing iron (II) sulphate heptahydrate (FeSO_4_·7H_2_O) and (PEI, *M*w = 25 kDa) is added dropwise over a basic solution with a mild oxidant under continuous mechanic stirring. Both solutions are mixed in a three-necked flask where a flux of N_2_ was bubbled. The synthesis temperature is maintained at 90 °C for 24 h, and after this time the PEI–MNPs are collected using a magnet and washed with deionized water several times to maintain the physiological pH.

All chemicals used for the synthesis were purchased from Merck KGaA (Darmstadt, Germany).

### 2.3. Laccase Immobilization

The immobilization of Laccase was carried out as previously reported [36]. Twenty-five milligrams of laccase from *Trametes versicolor* (EC 1.10.3.2) were dissolved in 3.0 mL sodium citrate buffer solution (10^−3^ mol L^−1^, pH 5.0) in the presence of 534 mg acrylamide and then, 466 mg polyethylene glycol dimethacrylate 750 and 2.4% 1-[4-(2-hydroxyethoxy)-phenyl]-2-hydroxy-2-methyl-1-propane-1-one (Irgacure 2959, with a maximum absorption at around 275 nm) were added as crosslinker and photo-initiator, respectively. After pouring in a reaction cell consisting of two 10 × 10 cm^2^ glass plates brought together by binder clips and separated with Teflon spacers (thickness 1.6 mm), the solution was polymerized by using a high pressure mercury lamp (HPK 125, Philips, Amsterdam, The Netherlands, 10 mW cm^−2^, wavelength 275 nm, irradiation time 10 min). The resulting hydrogel was freed from unreacted species by washing with distilled water and then dried for 12 h in an oven under vacuum at 40 °C.

Irgacure 2959 was from BASF, Ludwigshafen, Germany, all other chemicals from Merck KGaA (Darmstadt, Germany).

### 2.4. Synthesis and Characterization of the C@HSA Conjugate

C@HSA was synthesized via enzyme catalysis as follows: 500 mg HSA and 60 mg curcumin (CUR) were dissolved in 20 mL phosphate buffer solution (10^−3^ mol L^−1^, pH 6.8)/DMSO mixture 75/25 by volume and reacted by adding immobilized laccase (500 mg, 0.23 U) at 37 °C under 70 rpm for 12 h. The conjugate was purified by dialysis process (dialysis tubes of 6–27/32″ Medicell International LTD (Liverpool, UK), MWCO: 12,000–14,000 Da) and dipped into a glass vessel containing distilled water at room temperature for 72 h, until complete removal of unreacted CUR in the washing media was confirmed by high-pressure liquid chromatography (HPLC) analysis. The resulting solutions were frozen and dried with a freeze drier (Micro Modulyo, Edwards Lifesciences, Irvine, CA, USA) to afford vaporous solids.

The HPLC analysis conditions were: Jasco PU-2089 Plus liquid chromatography equipped with a Rheodyne 7725i injector (fitted with a 20 μL loop), a Jasco UV-2075 HPLC detector operating at 420 nm, Jasco-Borwin integrator (Jasco Europe s.r.l., Milan, Italy), and a Tracer Excel 120 ODS-A column particle size 5 μm, 15 × 0.4 cm (Barcelona, Spain), mobile phase consisting of methanol at a flow rate of 1.0 mL min^−1^ [37].

The (2,4,6)-trinitrobenzenesulfonic acid (TNBS) assay was used to determine the free amino groups in HSA and C@HSA, and thus to indirectly calculate the functionalization degree expressed as mg of CUR per g of conjugate [38]. Briefly, 0.5 mL 2,4,6-trinitrobenzenesulfonic acid solution (0.01% *w*/*v*) were added to 1.0 mg NaHCO_3_ (0.1 mol L^−1^, pH 8.5) containing HSA or C@HSA (2.0 mg mL^−1^) and, after mixing in the dark, placed in a water bath at 37 °C for 4 h under shaking. Then, SDS (50 μL, 0.35 mol L^−1^) and HCl (25 μL, 1 mol L^−1^) were added to terminate the reaction the absorbance measured at 335 nm on the V-530 Jasco UV–Vis spectrophotometer operating with 1.0 cm quartz cells (Jasco Europe, Milan, Italy). The amount (mg) of CUR in C@HSA was calculated according to Equation (1):

(1) $$mg_{CUR}=\frac{A_{0}-A_{1}}{A_{0}}\times n_{NH_{2}}^{0}\times MW_{CUR}$$

Here, $n_{NH_{2}}^{0}$ refers to the amount of NH_2_ groups in the HSA, $MW_{CUR}$ the molecular weight of CUR, and *A*_0_ and *A*_1_ the absorbance of HSA and C@HSA, respectively.

All chemicals were from Merck/Sigma Aldrich, Germany.

### 2.5. Synthesis and Characterization of Nanohybrid C@HSA-MNPs@rGO

For the synthesis of the nanohybrid C@HSA-MNPs@rGO, 45 mg C@HSA were added to 2.5 mL of MNPs@GO suspension synthesized by mixing 0.2 mL MNPs and 5.0 mg GO [39], and the resulting solution was stirred at 37 °C for 12 h. The nanohybrids were recovered as dark powder after freeze-drying process (Micro Modulyo, Edwards Lifesciences, Irvine, CA, USA).

Control nanoconjugates and nanocarriers were prepared in the same conditions employing HSA instead of C@HSA (HSA-MNPs@GO) or in the absence of MNPs (C@HSA-rGO and HSA-GO).

Scanning electron microscopy (SEM) images were obtained using a NOVA NanoSEM 200 (Thermo Fisher Scientific, Hillsboro, OR, USA) with an acceleration voltage of 15 kV after depositing samples onto self-adhesive conducting carbon tape (Plano GmbH, Wetzlar, Germany).

Thermogravimetric analysis (TGA) was performed with an SDT Q600 (TA Instruments, Wetzlar, Germany). Measurements were conducted in a nitrogen atmosphere (flow of 10 mL min^−1^), with an initial sample weight of ∼10 mg in the temperature range 25–600 °C at a heating rate of 10 °C min^−1^.

The magnetization curves as a function of field and temperature were obtained using a commercial Superconducting Quantum Interference Device (SQUID, model MPMS by Quantum Design, Inc., San Diego, CA, USA). Measurements were performed within the 5 ≤ *T* ≤ 300 K temperature range, and applied magnetic fields up to *H* = 3989 kA/m (5 Tesla).

### 2.6. In Vitro Releasing Tests

Here, 0.5 mg DOX were stirred with 50 mg C@HSA-MNPs@rGO, HSA-MNPs@GO, and C@HSA-rGO in 5.0 mL distilled water for 24 h and then the loaded nanohybrids recovered after drying under vacuum. The release experiments were performed in phosphate buffered saline (10^−3^ mol L^−1^) at pH 7.4 or 5.0. Briefly, 15.0 mg loaded nanohybrid was dispersed into 1.5 mL phosphate buffered saline at selected pH into a dialysis bag (MWCO: 12,000–14,000 Da), and dialyzed against 13.5 mL of the corresponding buffer. At predetermined time intervals, the amount of DOX in the releasing media was determined by UV–Vis on a Jasco V-530 UV/Vis spectrometer (Jasco Europe s.r.l., Milan, Italy) at 496 nm. From the calibration curves of DOX sketched in PBS (pH 7.4 and pH 5.0, respectively), the cumulative amount of drug released was calculated using Equation (2):

(2) $$\frac{M_{t}}{M_{0}}=\frac{M_{t}}{M_{total}}$$

where *M_t_* and *M*_0_ are the amounts of drug in solution at time *t* and loaded into the carrier, respectively.

### 2.7. Cell Viability Assay

Human neuroblastoma SH-SY5Y (ATCC® CRL-2266™) cells were employed to assess the cytotoxicity effect. Cells were grown in DMEM medium supplemented with 1% L-glutamine and 10% fetal bovine serum, routinely maintained in culture at 37 °C and 5% CO_2_ and regularly screened to ensure the absence of mycoplasma contamination using the MycoAlert MycoPlasma Detection Kit (Lonza, Switzerland).

For cytotoxic experiments, cells were plated in clear transparent 96-well plates at optimized cell densities of 10^4^ cells per well. To ensure cell attachment, cells were seeded 24 h before being treated. Cells were treated with free DOX and DOX loaded nanohybrids for 72 h. To determine the effect of the treatments on the cell viability, we counted the viable cell by using the Trypan Blue exclusion method as previously described [40]. All the cell viability assays have been conducted in the presence of 10% serum.

Finally, to evaluate the possibility to spatially control the DOX activity, viability experiments were performed by treating 250 × 10^3^ cells seeded in a 35-mm petri dish with DOX loaded on either C@HSA-MNPs@rGO (DOX-C@HSA-MNPs@rGO) or C@HSA-rGO (DOX-C@HSA-rGO) for 72 h under the effect of a magnetic field generated by a permanent magnet (100 G).

### 2.8. Cell Internalization Studies

To prepare the samples for TEM analysis, the human neuroblastoma cells SH-SY5Y were seeded and incubated with C@HSA-MNPs@rGO. Cell internalizations study was carried out after 6 and 24 h. After co-incubation, the medium was removed and the cells were detached and fixed with 2% glutaraldehyde solution at 4 °C. After washing, cells were dehydrated with increasing concentrations of acetone and embedded in a solution (50:50) of epoxy resin. Then the EPOXI resin was cut in 70-nm thin slices. The samples were analyzed by transmission electron microscopy (TEM) using a FEI Tecnai T20 microscope operating at 200 keV.

### 2.9. Statistical Analysis

Three experiments were carried out in triplicate. Values were expressed as means ± standard error of the mean (SEM). For viability assay, statistical significance was assessed by one-way analysis of variance followed by post-hoc comparison test (Tukey’s test). Significance was set at *p* < 0.01.

## 3. Results

**Figure 1:**
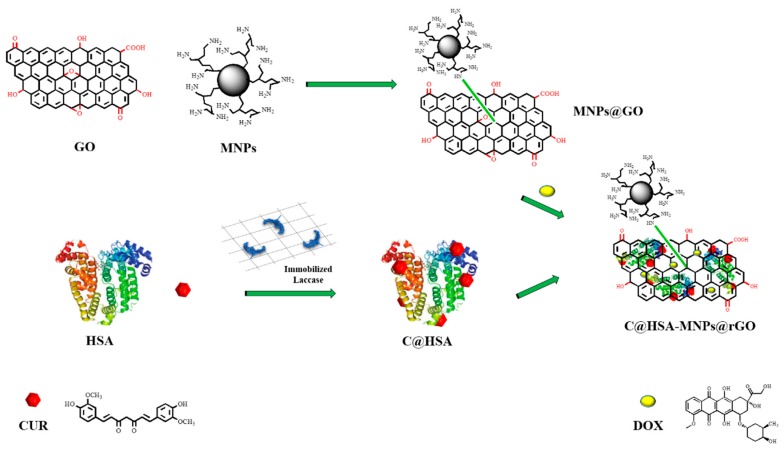
Schematic representation of the synthesis of nanohybrid C@HSA-MNPs@rGO.

The multifunctional nanohybrid described here is composed of C@HSA conjugate, GO, and MNPs, each addressing a specific requirement to develop a cancer therapy that could efficiently deliver DOX to neuroblastoma SH-SY5Y cells (Figure 1).

The synthetic procedure involved at first the synthesis of the inorganic (MNPs@GO) and organic counterparts (C@HSA), and subsequently their coupling for the obtainment of the nanohybrid C@HSA-MNPs@rGO.

The MNPs@GO system obtained via GO functionalization with polyethyleneimine polymer (PEI)-coated magnetic nanoparticles (PEI-MNPs) exploited the ability of PEI to covalently react with GO sheets via a “grafting to” process to produce highly stable nanohybrids [39]. For this purpose, MNPs were synthesized by a previously reported procedure [19] based on the oxidation of Fe(OH)_2_ by nitrate in basic aqueous media in the presence of branched PEI (25 kDa). By using this approach, we obtained PEI-MNPs with an average particle size of 25 ± 5 nm, composed of a thin layer (7–9 Å) of PEI coating around the Fe_3_O_4_ core, showing high stability to aggregation, and the presence of functional groups on the particle surface suitable for further modification [35]. Then, the MNPs@GO system was obtained by mixing GO (average size of 100 nm), synthesized by a modified Hummer’s method [34], with PEI-MNPs under vigorous stirring at room temperature.

**Figure 2:**
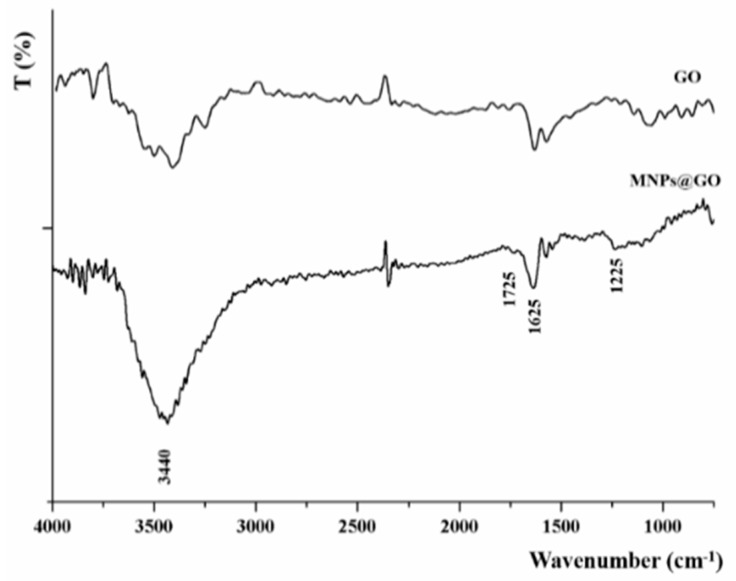
FTIR spectra of GO and MNPs@GO.

The FTIR spectrum of MNPs@GO (Figure 2) showed the retention of the typical GO peaks at 3440 (O–H stretching), 1725 (stretching COOH), and 1625 (stretching C=C) cm^−1^, with the disappearance of the stretching peak of epoxy C–O groups located at 1225 cm^−1^ invoked as a confirmation of the successful formation of MNPs@GO system due to the reaction with the amine groups of PEI. The presence of MNPs on GO surface was further confirmed by the increase in the intensity of the band located at around 3400 cm^−1^, which can be mainly ascribed to the N–H stretch vibration of PEI, and O–H stretching of water molecules bound to MNPs.

It is well known that for the preparation of effective nanocarriers, a surface modification of GO sheets is required to overcome their tendency to aggregate in physiological environment [15]; for this purpose different types of polymeric materials have been proposed as coating element [41]. Here, we coated MNPs@GO with a C@HSA conjugate to take advantage of the excellent biocompatibility and non-immunogenicity of HSA [42,43], and the biological activity of curcumin, a naturally occurring polyphenol with pronounced anticancer activity [17], with the aim to fabricate a functional nanohybrid (C@HSA-MNPs@rGO), in which the anticancer activity is related to both the controlled release of loaded drug and the carrier itself [29]. The covalent conjugation of CUR to HSA improved its stability within the physiological compartments [44]. This is a quite important point considering that improved aqueous solubility and stability of CUR are essential for its potential clinical application [45]. Furthermore, it is important to note that the CUR dose remain unmodified during the vectorization process, since no CUR delivery occurred within 24 h (data not shown).

**Figure 3:**
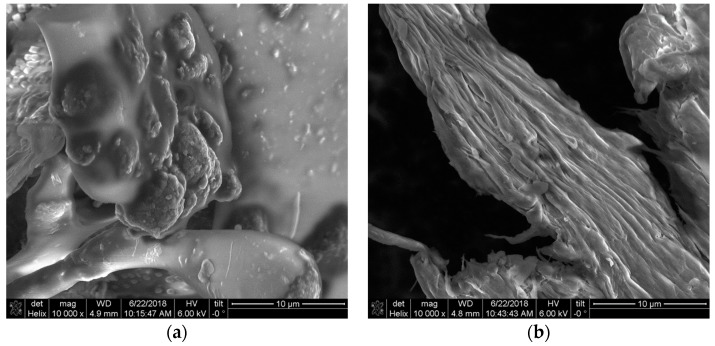
SEM pictures of nanohybrid C@HSA-MNPs@rGO.

The C@HSA conjugate was synthesized via a fully green process involving the use of a solid biocatalyst previously developed and characterized and consisting of laccase immobilized into acrylate hydrogel film with a functionalization degree of 91 mg of CUR per gram of conjugate, as per combined UV–VIS analysis and TNBS assay. Moreover, the hypsochromic shift recorded comparing the UV–VIS spectra of C@HSA and CUR from 420 to 347 nm was used as a confirmation of the covalent conjugation of the polyphenols to the protein backbone [46]. The reaction mechanism for the coupling of nanocarrier MNPs@GO with nanoconjugate C@HSA consisted of a redox reaction between the organic and inorganic counterparts, where the polyphenol moieties were converted to the corresponding oxidized quinone forms with adequate potential to reduce the oxygen-rich functionalities of GO, obtaining a reduced graphene oxide (rGO) nanohybrid (C@HSA-MNPs@rGO) [47,48,49,50]. The effective formation of nanohybrid C@HSA-MNPs@rGO was verified by SEM investigation (Figure 3a,b), where it is clear that the rGO nanosheets were homogeneously coated by the protein film.

**Figure 4:**
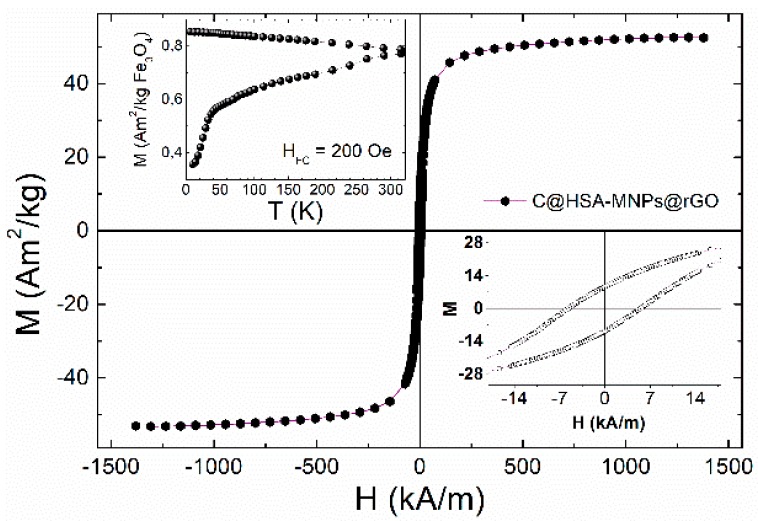
Main panel: Magnetization M data vs. applied field *H* of nanohybrid C@HSA-MNPs@rGO at room temperature. Upper left panel: Zero-field-cooled and field-cooled M(T) curves at applied field *H* = 15.92 kA/m. Low-right panel: Magnification of the low-field region of the M(H) hysteresis loop, showing the measurable coercive field at *T* = 295 K as a reflect of the blocked state of the magnetic nanoparticles within the nanohybrid.

The magnetic response of the nanohybrids was explored by M(T) and M(H) data (Figure 4).

The temperature dependence M(T) using zero-field-cooling and field-cooling protocols showed no maximum in the ZFC curves corresponding to a blocking temperature, as expected due to the fact that single-domain magnetic cores of magnetite have an average size of over 40 nm (as observed in TEM images, see later). These Fe_3_O_4_ nanoparticles are expected to be blocked [51,52], given their size and value of magnetocrystalline anisotropy that yield an anisotropy relaxation energy barrier larger than thermal energy at room temperature. The hysteresis loops at room temperature showed saturation magnetization values (*M*_S_) = 48–52 Am^2^/kg, somewhat lower than the bulk Fe_3_O_4_. The observed lowering of the *M*s when compared to the bulk values of 80–85 Am^2^/kg is related to a low crystallinity of the MNPs that in turn is probably originated in the low temperature of the hydrothermal route used in this work [35,53]. The existence of a coercive field of *H*_C_ = 5.82 kA/m at room temperature supported the hypothesis that these magnetic cores are magnetically blocked.

**Figure 5:**
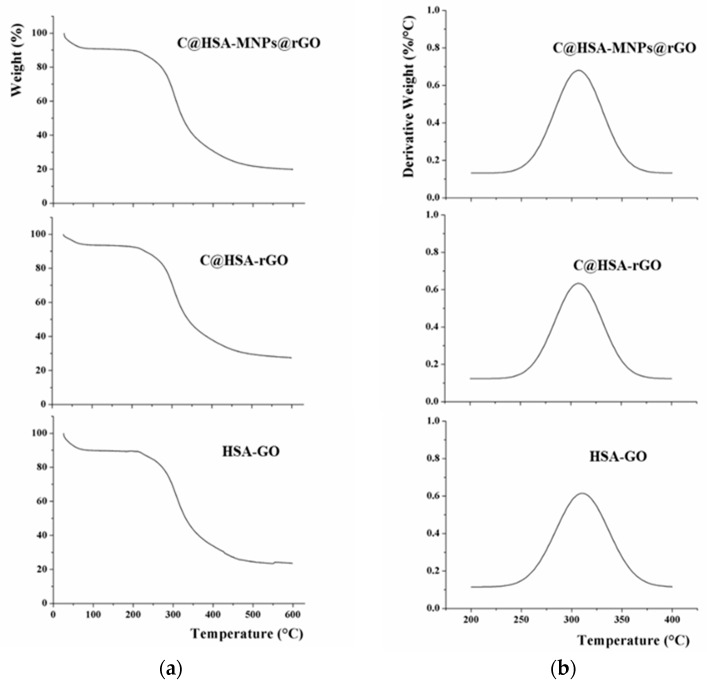
TGA (**a**) and DTG (**b**) curves of C@HSA-rGO, HSA-GO, and C@HSA-MNPs@rGO.

TGA analyses (Figure 5a) were performed to check the thermal stability of the final nanohybrids. At first, HSA-GO was employed as control sample, and the effect of each component was highlighted by measuring C@HSA-rGO and C@HSA-MNPs@rGO. The presence of CUR residues was found to enhance the thermal stability of C@HSA-rGO [54], with a lower degradation (73% vs. 77%) recorded at 600 °C (Figure 5a). On the other hand, the higher amount of organic counterpart (PEI coating of PEI-MNPs) resulted in a lower thermal stability of C@HSA-MNPs@rGO (degradation of 80%). The same trend was recorded when considering the weight loss at the degradation temperature, located at around 310 °C for all samples (see the derivative thermogravimetric analysis, DTG curve, Figure 5b), with the degradation values of 38 (HSA-GO), 33 (C@HSA-rGO), and 40% (C@HSA-MNPs@rGO).

### 3.1. In Vitro Doxorubicin Release

**Figure 6:**
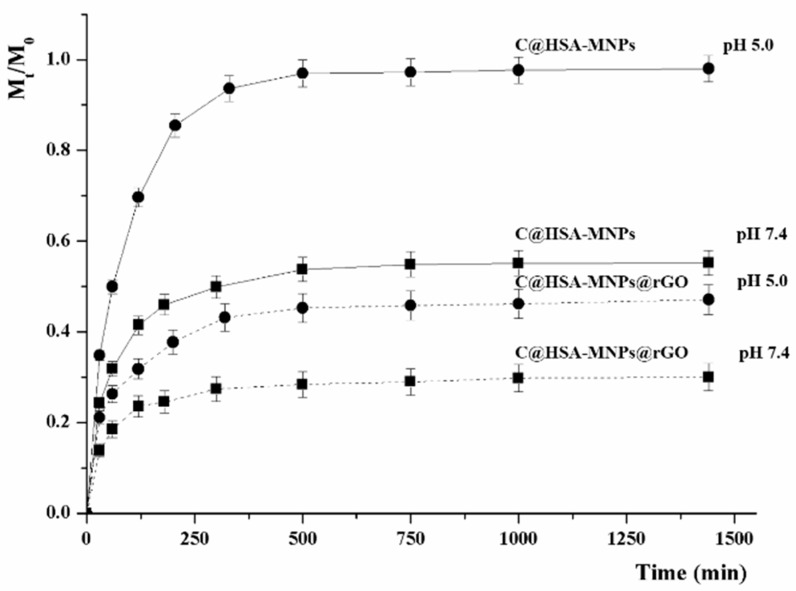
pH-responsive DOX release profile (Mt/M0) from C@HSA-MNPs (solid line) and C@HSA-MNPs@rGO (dashed lines) at pH 5.0 (●) and 7.4 (■).

The nanohybrid C@HSA-MNPs@rGO was designed as DOX delivery vehicle, employing a drug to rGO ratio (by weight) of 12.5%. Release experiments were performed at pH 5.5 and 7.4, simulating the endosomal pH of cancer cells and the normal physiological pH, respectively [55], and, to highlight the importance of rGO within the nanocarrier, the results were compared with those obtained when C@HSA-MNPs, not containing rGO, were employed (Figure 6).

Assuming that the amount of DOX in the release media is a result of the partition between the carrier and the surrounding environments, the application of a mathematical modelling proposed in literature allowed the determination of key parameters describing this phenomenon according to reversible first- and second-order kinetics (Equations (3) and (4)) [56]:

(3) $$\frac{M_{t}}{M_{0}}=F_{max}\left(1-e^{-\left(\frac{k_{R}}{F_{max}}\right)t}\right)$$

(4) $$\frac{M_{t}}{M_{0}}=\frac{F_{max}\left(e^{2\left(\frac{k_{R}}{\alpha }\right)t}-1\right)}{1-2F_{max}+e^{2\left(\frac{k_{R}}{\alpha }\right)t}}$$

*F_max_* is the maximum value of relative release (*M*_t_/*M*_0_), *k*_R_ the release rate constant, while the physicochemical affinity of the drug between the carrier and solvent phases (α) can be determined according to the following Equation (5):

(5) $$\alpha =\frac{F_{max}}{1-F_{max}}$$

Both Equations (3) and (4) can be used to calculate the time required for reaching 50% of *F_max_* ($t_{1/2}^{R}$) (Equations (6) and (7)), respectively:

(6) $$t_{1/2}^{R^{1}}=\frac{F_{max}}{k_{R}}ln2$$

(7) $$t_{1/2}^{R^{2}}=\frac{\alpha }{2kR}\ln\left(3-2F_{max}\right)$$

The experimental data were well fitted by either Equation (3) or (4) (*R*^2^ > 0.95, Table 1), with predominant reversible first-order kinetics.

Figure 6 clearly showed pH-sensitive behavior, with the reduction of pH values carrying out to higher amount of released drug (*F_max_* of 45% and 28% at pH 5.5 and 7.4, respectively, see Table 1). Compared to physiological environments, at lower pH value, indeed an easier DOX protonation occurred, with a reduction of the hydrophobic-driven interactions between drug and carrier (*α* moved from 0.39 to 0.82), somehow fastening the DOX dissolution (*k*_R_ increases from 0.50 to 0.60 and $t_{1/2}^{R}$ decreases from 52 to 39 min). However, at both pH values, the amount of DOX in the surrounding environments was always lower than 50%, highlighting the strong ability of rGO to load and carry drug molecules with high affinity [57]. The low DOX release at physiological pH is expected to dramatically reduce the drug side effects, thus enhancing the therapeutic index. This result underlines the importance of rGO into the nanocarrier, with C@HSA-MNPs showing a lower drug to carrier affinity (highest α values at both pH), with 53% of DOX released at pH 7.4, increasing up to 97% at acidic pH.

More detailed information about the release mechanisms can be obtained by applying the mathematical model proposed by Avrami (Equation (8)) [58], fitting well with the experimental data (*R*^2^ values > 0.92):

(8) $$\frac{M_{t}}{M_{0}}=1-e^{-kt^{n}}$$

*n* and *k* are the Avrami parameter and release rate constant, respectively and were calculated by rewriting of Equation (8) by double logarithm (Equation (9)) and plotting of ln(ln(1 − *X*)) vs. ln *t*.

(9) $$\ln\left(-\ln\left(1-\frac{M_{t}}{M_{0}}\right)\right)=\ln k+n\ln t$$

The recorded *n* values for C@HSA-MNPs@rGO (Table 1) allowed for hypothesizing that the release kinetics were mainly diffusive (*n* close to 0.54). Interestingly, the carrier prepared in the absence of rGO (C@HSA-MNPs) showed a similar behavior at pH 7.4, while under acidic conditions a first-order kinetic was invoked to explain the higher DOX release.

The effect of rGO and pH on the releasing rate was better highlighted by the introduction of the half-life release time obtained when *M_t_*/*M*_0_ is 0.5 ($t_{1/2}$), according to Equation (10).

(10) $$t_{1/2}=e^{\left(\frac{-\ln k-0.367}{n}\right)}$$

The model reflected the experimental fact that, in the absence of rGO, faster release was detected in acidic ($t_{1/2}$ of around 60 min) compared to neutral ($t_{1/2}$ of around 290 min) conditions, while the presence of GO dramatically slowed the release and the $t_{1/2}$ values were found to be outside of the experimental range of 24 h. Finally, for both models, it should be pointed out that the presence of CUR did not significantly interfere with the release kinetics (*p* > 0.05), with the release profiles of HSA-MNPs and HSA-MNPs@GO being closer to C@HSA-MNPs and C@HSA-MNPs@rGO, respectively (data not shown).

### 3.2. Evaluation of Cytotoxic Activity

**Figure 7:**
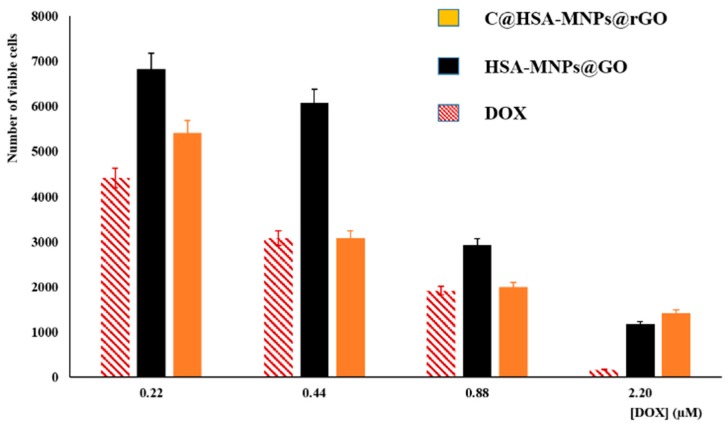
SH-SY5Y viability after treatment with free and loaded DOX on HSA-MNPs@GO and C@HSA-MNPs@rGO.

The cytotoxic activity of DOX after loading into C@HSA-MNPs@rGO nanohybrids (DOX to carrier ratio of 12.5% by weight) was tested on neuroblastoma SH-SY5Y cells (Figure 7). As a control sample, we employ DOX-loaded HSA-MNPs@GO to highlight the effect of the functional coating on the nanohybrid structure.

At first, free drug was tested in the range of 0.22–2.20 µM, showing a typical dose-dependent response. Then, empty carriers showed no toxicity in the tested concentrations (10.2 to 102 µg mL^−1^), with cell viability values (%) higher than 95% in all cases (data not shown).

**Figure 8:**
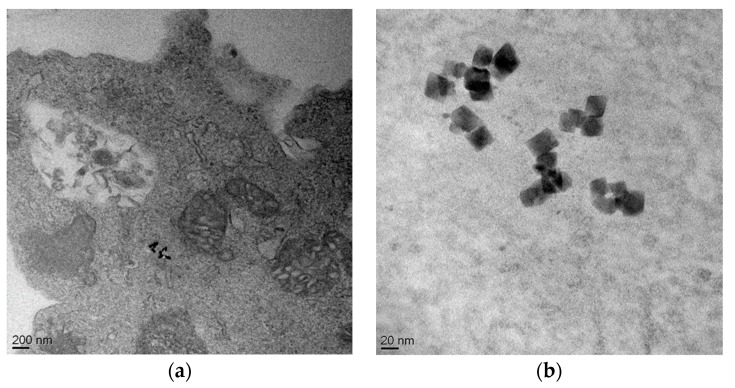
TEM images of SH-SY5Y after incubation with C@HSA-MNPs@rGO, proving the presence of nanohybrid within the cytoplasm.

Performing the viability assays with loaded systems at equivalent DOX concentrations, the effect of the nanohybrid was evident. The high affinity of GO for the anticancer therapeutic resulted in a significant reduction of the cytotoxic effect when HSA-MNPs@GO was employed as carrier. This is in accordance with the literature data proving that a slower release of DOX from nanoparticule delivery system is associated with a reduction of toxicity of loaded vs. free DOX at equivalent concentrations [59,60,61,62]. While free DOX penetrated the cell membrane through passive diffusion, comparatively long time was required for loaded DOX due to strong π–π stacking and endocytosis-mediated cytosolic sustained release [63,64,65]. In our experimental conditions, the ability of nanohybrid to cross the cell membrane is proved by TEM analysis (Figure 8), showing the presence of nanohybrids within the cytoplasm of SH-SY5Y cells.

**Figure 9:**
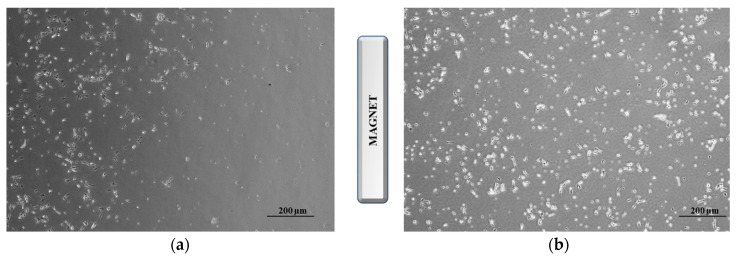
Optical microscope image SH-SY5Y incubated with DOX-C@HSA-MNPs@rGO (**a**) and DOX-C@HSA-rGO (**b**) under the effect of a permanent magnet.

To further improve the therapeutic outcome, we performed additional experiments employing similar carriers but functionalized with CUR (C@HSA-MNPs@rGO). In these experiments the well-known synergistic activity between the polyphenol species and DOX [66] enhanced the activity of the released drug, with the cytotoxicity becoming similar to the free DOX at 0.44 and 0.88 µM (Figure 7). The maintenance of the DOX anticancer efficiency, combined with both the selective release capability in acidic conditions and the magnetic properties, makes the proposed C@HSA-MNPs@rGO nanohybrid a promising carrier for a targeted therapy. It can be hypothesized that the carrier is able to spatially control the DOX anticancer activity being selectively attracted from a magnetic field. To confirm this finding, we incubated 250 × 10^3^ cells seeded in a 35-mm petri dish with DOX loaded on either C@HSA-MNPs@rGO or C@HSA-rGO for 72 h under the effect of a magnetic field generated by a permanent Nd-Fe-B magnet. For DOX-C@HSA-MNPs@rGO, the results showed a clear targeted cell death at the region close to the magnet, as a consequence of the increased local concentration of the drug by MNPs. Concurrently, no relevant toxicity was observed on the opposite region of the petri dish, where the magnetic forces were negligible (Figure 9a). The same experiment was performed with DOX-C@HSA-rGO as a control for the magnetic field effects, since the absence of the MNPs makes the compound not responsive to the magnetic field, which results in absence of any spatial control of the drug activity (Figure 9b).

Our results have proved the potential applicability of DOX-C@HSA-MNPs@rGO in the vectorization of DOX to neuroblastoma cells due to their capability to be selectively attracted from a magnetic field. These nanoparticles can potentially accumulate in tumors as a consequence of the enhanced permeability and retention (EPR) effect, and enable preferential drug release in response to the decreased tissue pH of either the extracellular (e.g., hypoxia) or intracellular (e.g., endosomes) tumor compartments [67]. Future experiments will be performed for evaluating the pharmacokinetics profiles with or without magnetic field, the anticancer activity, and the theranostics (e.g., MRI imaging) properties of the nanohybrid in suitable orthotopic neuroblastoma xenograft models.

## 4. Conclusions

We presented experimental evidence that the novel C@HSA-MNPs@rGO is a capable nanoplatform to provide spatial control for the release of cytotoxic agents such as DOX, due to the combined unique features of GO, MNPs, and C@HSA conjugates. While GO was responsible for the observed high loading capacity, the MNPs provided remote-actuation capabilities to deliver the nanohybrids to specific target sites. Moreover, the C@HSA conjugate coating acted synergistically with the DOX to increase the therapeutic effects on cancer cells.

The synthetic strategy consisted in two steps: C@HSA and MNPs@GO were preliminary fabricated via enzyme catalysis and chemical coupling, respectively, and then assembled to form the final nanohybrid C@HSA-MNPs@rGO. The physico-chemical characterization of the nanohybrids regarding their morphological, thermal, and magnetic properties showed the correct assembly of the components as well as the magnetic responsiveness expected for a targeted delivery vehicle for DOX. The DOX release profile was found to be tunable by pH, as a consequence of the modulation of the drug to carrier affinity, whereas the in vitro viability assays clearly showed the enhancement of cytotoxic activity due to the presence of CUR moieties within the nanohybrid coating. The observed levels of nanohybrid internalization by SH-SY5Y cells, together with magnetic actuation by external magnetic fields to increase local concentration at targeted sites, makes this nanohybrid a promising carrier for neuroblastoma treatment.

## Figures and Tables

**Table 1:** R^2^ values and kinetic parameters.

Mathematical Model	Parameter	C@HSA-MNPs		C@HSA-MNPs@rGO	
		pH			
		2.0	7.0	2.0	7.0
$\frac{M_{t}}{M_{0}}=F_{max}\left(1-e^{-(k_{R}/M_{max})t}\right)$	***R*^2^**	0.9935	0.9747	0.9563	0.9731
	***Fmax***	0.97	0.53	0.45	0.28
	***α***	32.33	1.13	0.82	0.39
	***k*_R_ (10^−2^)**	1.13	0.79	0.60	0.50
	$t_{1/2}^{R^{1}}$ **(min)**	60	47	52	39
$\frac{M_{t}}{M_{0}}=\frac{F_{max}\left(e^{2\left(\frac{k_{R}}{\alpha }\right)t}-1\right)}{1-2F_{max}+e^{2\left(\frac{k_{R}}{\alpha }\right)t}}$	***R*^2^**	0.9717	0.9765	0.9527	0.9639
	***Fmax***	0.98	0.53	0.45	0.28
	***α***	49.00	1.13	0.82	0.39
	***k*_R_ (10^−2^)**	1.87	0.81	0.58	0.44
	$t_{1/2}^{R^{2}}$ **(min)**	52	46	52	39
$\frac{M_{t}}{M_{0}}=\;1-e^{-kt^{n}}$	***R*^2^**	0.9946	0.9933	0.9888	0.9248
	***K* (10^−2^)**	2.90	8.46	7.28	6.14
	***n***	0.78	0.37	0.35	0.29
	$t_{1/2}^{A}$ **(min)**	58	294	#	#

# outside experimental range.
